# Economic Research on Ethanol Feed-Use Coproducts: A Review, Synthesis, and Path Forward

**DOI:** 10.3390/ani14111551

**Published:** 2024-05-24

**Authors:** Elliott Dennis, Daniel Gertner, Galen Erickson

**Affiliations:** 1 Department of Agricultural Economics, University of Nebraska—Lincoln, Lincoln, NE 68583, USA; 2 The Good Food Institute, Washington, DC 20090, USA; gertner.dan@gmail.com; 3 Department of Animal Science, University of Nebraska—Lincoln, Lincoln, NE 68583, USA; gerickson4@unl.edu

**Keywords:** economic, ethanol, livestock, coproduct, distiller grains, corn oil, synthesis review

## Abstract

**Simple Summary:**

Simple Summary: During the ethanol creation process, ethanol and ethanol co-products are created. Ethanol plants take these co-products and market them in a secondary market. While originally just additional revenue streams, coproducts have evolved into valuable commodities themselves. Analyzing existing research, the study finds that most of the current economic understanding is for distillers’ grains, a common coproduct used in animal feed. Newer coproducts like pelletized or high-protein grains are less studied. Moreover, alternative uses for coproducts such as plastics and CO_2_ have remained largely unexplored. The research underscores the need for further investigation to optimize coproduct utilization and inform future decision-making. We suggest focusing on the economic pricing, marketing, and regulatory structure of newer ethanol co-products to add additional value to the ethanol industry.

**Abstract:**

During the mid-2000s to the early 2010s, the domestic ethanol industry witnessed substantial growth, with ethanol coproducts emerging as vital elements for plant profitability and livestock feeding. Initially serving as supplementary revenue streams, coproducts from ethanol production have evolved into diverse value-added offerings, bolstering revenue streams, and sustaining profit margins. This study reviews existing economic research on ethanol coproducts, detailing methodologies, product focus, and research locations. Initially gathering 972 articles from 9 databases, 110 articles were synthesized. We find that most studies primarily examined the growth and future of the ethanol industry with a limited focus on specific coproducts. Feed-use distillers’ grains, especially dried distillers’ grains, were the most widely published while newer coproducts like pelletized, de-oiled, and high-protein distillers’ grains were relatively understudied. Non-feed-use products were notably overlooked, highlighting the need for exploration beyond conventional applications. The evolving market landscape for ethanol co-products has surpassed published academic understanding of the economic tradeoffs necessitating further research into product dynamics, pricing, marketing, market structures, and regulatory frameworks. This highlights and underscores the importance of investigating value-added grains across diverse commodities and geographic contexts to inform strategic decision-making and policy formulation.

## 1. Introduction

**Figure 1:**
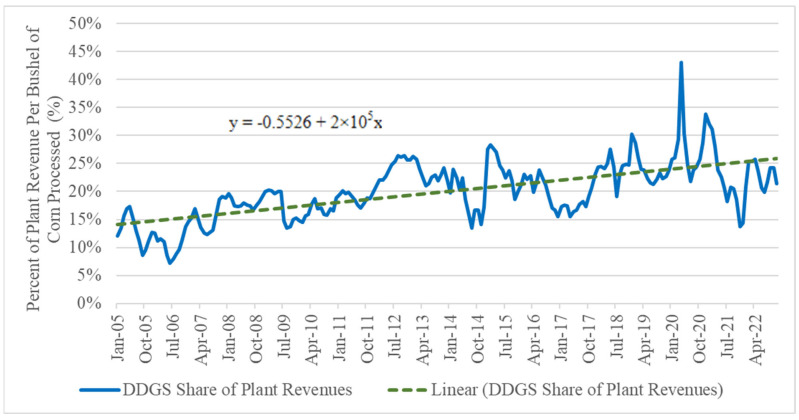
Monthly DDGS percent of ethanol plant revenue per bushel of corn processed, 2005–2022. Source: Authors’ calculations using Iowa Ethanol Plant Profitability Model (https://www.extension.iastate.edu/agdm/energy/html/d1-10.html (accessed on 5 May 2023)).

During the domestic ethanol boom of the mid-2000s to early 2010s, feed-use coproducts, primarily in the form of distiller grains and corn oil grew to play a crucial role in both ethanol plant profitability and livestock feeding rations. Revenues from selling feed-use coproducts have helped maintain plant profit margins despite demand volatility for ethanol and the supply of coproducts. For example, revenue from distillers’ grains as a share of total plant revenue has risen from an average of 12% in 2005 to 22% in 2022 (Figure 1). During the ethanol plant closures and shutdowns during the COVID-19 pandemic, this share spiked to nearly 40% of ethanol plant revenues with a simultaneous decline in ethanol demand and rising demand for feed resources from local livestock producers. Including corn oil and high protein distillers’ grain products would likewise increase these revenues. 

One bushel of corn leads to, on average, 2.9 gallons of denatured fuel ethanol, 15.2 pounds of distillers’ grains, 0.8 pounds of corn oil, and 1.1 pounds of carbon dioxide [1]. This fact has led to a large focus on feed-use distiller grains as a way for ethanol plants to grow revenues and search for methods to extract additional value out of each product in the distillers’ grain space. Many ethanol plants have moved beyond the traditional dried, modified, and wet distillers’ grain offerings by developing new technologies to modify or refine distiller grains to extract more value. Innovations have included pelletized distillers’ grains, de-oiled distillers’ grains, high protein distillers’ grains, corn oil, and a variety of other products. These new products have further differentiated distillers’ grains and have helped to establish distiller-type feeds as goods with increasingly separate demand structures from the ethanol market. Many of these fundamental changes within both feed-use coproducts have occurred within the last 5–10 years potentially changing our current understanding and conclusions of feed-use ethanol coproducts. 

As a result, ethanol’s impact on regional and national economies inhabits a well-explored segment of the academic literature (see [2] for a review of relevant studies). Of the studies that include ethanol coproducts, the vast majority are related to the feed-use distillers’ grains long considered a secondary byproduct of biofuel production. Even among distillers’ grains, the majority of economic research focuses primarily on dried distillers’ grains. When other coproducts are analyzed–such as wet and modified wet distillers’ grains–the focus is often on how they vary or compare to dried distillers’ grains. Further, these impacts vary by livestock species as a protein substitute [3,4].

The purpose of this paper is to provide a comprehensive review that synthesizes the current economic research on feed-use ethanol coproducts and points to the need for more research. We specifically show how both physical markets have moved well beyond the current academic understanding of market products and structure. The primary focus for most published papers has been on the growth, impact, and future of the ethanol industry itself rather than conducting analyses specifically focusing on specific feed-use or non-feed-use coproducts. 

We detail, explain, and organize this paper around the production and use of distiller grains as follows. First, we provide additional context to the development and use of distillers’ grain production. Second, we detail the methods behind the collection, selection, and organization of the research included in this review. Third, the ethanol coproduct economic research is synthesized into one of six general categories. Fourth, given the findings from the synthesis section, we justify some future paths forward and their potential implications for ethanol feed-use coproducts. The fifth section summarizes and concludes the paper.

## 2. Distillers’ Grains Market Development

### 2.1. Ethanol Plant Coproduct Production

Ethanol plants continually seek to market distillers’ grains as they are an inevitable byproduct of the ethanol production process. This comes in the form of finding new ways to market traditional distillers’ grains or by creating new value-added products. Traditional offerings include dried (DDG), modified wet (MDG), and wet distillers’ grains (WDG). Value-added products include dried distillers’ grains with solubles, pelletized distillers’ grains, de-oiled distillers’ grains, high-protein distillers’ grains, and a variety of additional products. In the absence of market demand for distillers’ grains, the coproducts must be disposed of by the plants, often at a sizeable cost, and at times can reduce ethanol production. By marketing distillers’ grains to livestock and poultry producers, ethanol plants monetize a necessary waste product, while livestock producers gain an often more affordable feed source, relative to corn, for livestock rations that can increase animal performance. 

Distillers’ grains are produced via the dry-grind ethanol production process as opposed to the wet milling process, the latter of which primarily results in corn gluten feed and other products/feed products. The dry-grind process aims to ferment the highest possible percentage of starch in the corn kernel. In this process, the entire corn kernel is processed and little is left to waste. The starch in the kernel is converted to sugars, then fermented to ethanol and carbon dioxide, while the remaining protein, lipids, fiber, minerals, and vitamins are converted into coproducts such as thin stillage and wet distillers’ grains. The thin stillage that is not recycled as processing water is concentrated into condensed distiller solubles (CDS) through evaporation. CDS are typically added back to distillers’ grains to create distiller grains with solubles (i.e., WDGS, MDGS, DDGS) [5]. They can also be fed as a separate byproduct to beef cattle [6] or can be used as a carrier in liquid feed supplements. 

### 2.2. Coproducts as a Feed Resource

Distillers’ coproducts have been produced for as long as the alcoholic distilling process itself, or since about 800 BCE [7]. Although the history of feeding those coproducts to animals is less robust, the practice still has a long history. The first documented study about feeding distillers’ grains to cattle was published in 1907. For much of the 20th century, the only distillers’ byproducts that existed on a large scale came from alcoholic beverage production. However, the proliferation of fuel ethanol production in the early 2000s created a new supply of distillers’ byproducts that were centered around areas of high agricultural production. 

Given distillers’ grains’ high crude protein content (25–35%), early market participants primarily considered distillers’ grains to be animal protein feed. Therefore, they were generally priced as an imperfect alternative to soybean meal, which has a crude protein content of 45–50%. As the supply of ethanol and distillers’ grains grew, the “sister” market shifted to the corn, rather than soybean, market. This was because distillers’ grains offered similar energy values to corn while excluding much of the starch, which was expended in the ethanol production process. The fact that distillers’ grains were produced from corn also meant that their pricing could be tied to the corn market. 

While both MDGS and WDGS offer greater feeding values than DDGS [8], their moisture contents and weights make them difficult to ship beyond a limited radius, making DDGS the most common form of distillers’ grains nationally and internationally. In most cases, all traditional distillers’ grains offer superior nutritional properties relative to dried rolled corn through a lower starch content, higher total digestible nutrients, and a higher content of crude protein but only for beef cattle [9]. These favorable properties and relative abundance of distillers’ grains in areas with high ethanol production rates—paired with a concerted effort by ethanol plants to market distillers’ grains as coproducts rather than by-products—helped form a market for distillers’ grains with a largely separate demand structure from the ethanol sector [10]. However, some industry participants have continued to argue that without the moisture and nutritional standard required of distiller grains produced, ethanol plants still sell them as a byproduct of ethanol production. 

When ethanol plants first ramped up production, they began selling distillers’ grains as feed to local livestock operations in the form of WDGS with 60–65% moisture to avoid the cost of drying or disposing of the byproduct. Ethanol plants then began investing more heavily in distillers’ grains drying technology to convert WDGS to DDGS which can be easily transported domestically and exported internationally. More recently, ethanol plants have developed a third product (MDGS) which increases its ability to be transported with improved handling while still allowing CDS to be mixed and used locally. A major reason for this development was to either (1) allow the ethanol plant to add all the CDS to distiller grains and not worry about selling the CDS at USD <10/ton or (2) resolve producer complaints about not all the CDS being added due to seeping of the product. Historically, ethanol plants will choose to produce one or two of these three products but then vary the quantity produced each month given market prices and current production contracts. 

### 2.3. Feed-Use Importance to Plant Profitability

Dried Distillers’ Grains with Solubles (DDGS), Modified Distillers’ Wet Grains (MDGS), and Distillers’ Wet Grains (WDGS) are the products that constitute the largest share of the coproduct revenue stream for most ethanol plants. According to estimates from the Iowa State ethanol plant profitability model, from 2005 to 2022 dried distillers’ grains’ percent of plant revenues per bushel of grain processed has increased from approximately 12% to 22% [11]. They also play a crucial role in maintaining profit margins and allow for diversified, value-added product offerings [12]. Weak crude oil prices paired with steady corn prices over the past half-decade decreased margins in the ethanol industry. To hedge against adverse price trends in the ethanol industry, ethanol plants have focused on diversifying their operations to produce feed products targeted at other livestock or aquaculture and increase the price [13].

## 3. Materials and Methods

### 3.1. Selection of Economic Distiller Grains Research

The economics behind traditional distillers’ grain products and newer products are explored in academic literature to differing extents. The purpose of this literature review is to identify, compile, synthesize, and analyze the existing economic research about distillers’ grains. Doing so provides insight into the distillers’ grain markets while identifying gaps in the literature and charting paths forward for future research. 

An open literature search was conducted using online databases including AgEconSearch, Agricola, Cambridge, CAB Abstracts and Global Health, EconLit, JSTOR, PubMed, Scopus, and Wiley Online Library. Articles in all databases were retrieved using the search string, (ethanol OR distiller*) AND (econ* OR pric*)”. All results were limited to publication years between 2000 and 2021. To refine retrieved results, the following database-specific adjustments were made: (1) AgEconSearch results were filtered by English-language articles, (2) CAB Abstracts and Global Health and JSTOR results were filtered by terms found in the abstract, (3) Cambridge results were filtered by “access”, and (4) Scopus results were filtered by the keyword “Economics”. No modifications were made to the Agricola, EconLit, or Wiley database searches.

**Figure 2:**
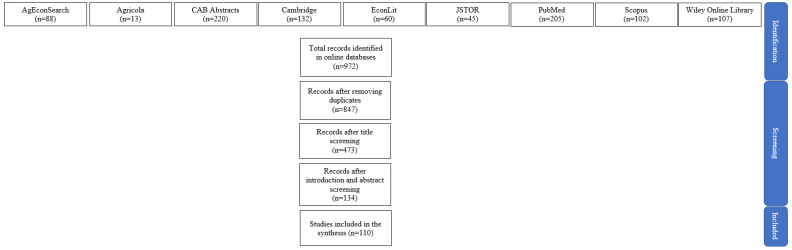
Search, filtering, and selection process for articles used in the synthesis review. Source: Authors’ compilation.

In total, 972 articles were retrieved across the 9 databases. After removing duplicate articles, 847 results remained. Article titles were then examined for English language and relevance to topics related to biofuels, grains, feed, livestock, and economic analysis. Only titles clearly unrelated to ethanol coproduct production (i.e., “Medication Use Safety During Care Transitions for Children with Medical Complexity”) were filtered out. Following the title analysis, 473 articles remained. Next, abstracts and introductions were read to determine their pertinence to the synthesis. This required the articles to discuss and analyze at least one feed distiller grain or ethanol feed-use coproduct. After screening abstracts and introductions, 134 articles remained. Articles that conducted at least one economic analysis related to ethanol feed coproduct markets, production, or demand were included in the final review. Economic analysis was broadly defined and meant to include basic cost–benefit analyses in addition to more complex econometric analyses. In total, 110 articles were included in this literature review and synthesis. Figure 2 provides a summary of the total article retrieval and screening process. 

### 3.2. Categorization of Economic Distiller Grains Research

Selected articles were classified by peer-reviewed publication status, the type of distiller grains examined, livestock type, other grain markets impacted by distiller grain use, and location of study. Common research topics between articles were identified and selected as the primary categories. Studies were then classified by which broad topic best fit the paper’s content. In situations where a paper could reasonably fit into multiple categories, papers were categorized by the primary analysis used in the article. Table 1 summarizes and categorizes all 110 papers used and Table S1 in the supplemental material provides the details for each specific paper. 

#### 3.2.1. Topics Covered

**Figure 3:**
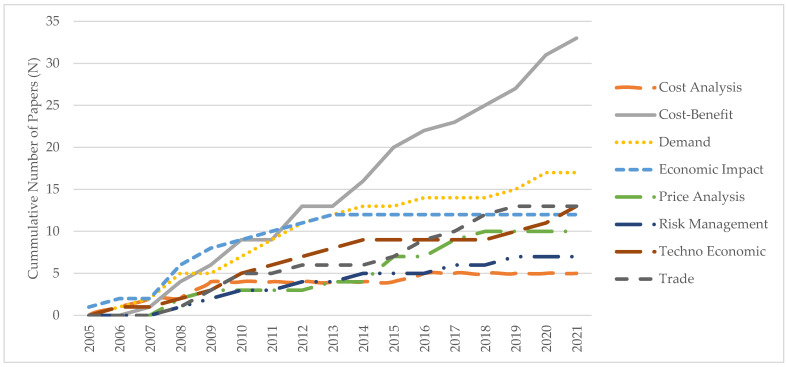
Cumulative number of articles in the synthesis review by primary method of analysis, 2005–2021. Source: Authors’ compilation. Note: There are a total of 110 articles in the synthesis review.

All papers were classified into one of eight topic categories based on the primary emphasis of the paper (see panel (a) of Table 1). Topic categories (papers) included cost analysis (5), cost–benefit analysis (33), demand analysis (17), economic impact analysis (12), price analysis (10), risk management (7), techno-economic analysis (13), and trade analysis (13). In cases where an article used more than one method of analysis, we categorized the paper by the primary analysis method used. Some methods of analysis have become more/less popular since distiller gains were first developed (see Figure 3). For example, papers employing cost analysis ranged from 2006 to 2016 whereas papers employing economic impact analysis ranged from 2005 to 2013. This suggests that some analyses have become more/less popular over time likely because many basic research questions in these categories were already explored by the mid-2010s. The cost–benefit analysis was the most popular form of analysis.

#### 3.2.2. Peer-Reviewed vs. Non-Peer-Reviewed

We classified papers as either peer- or non-peer-reviewed papers. Papers that came from academic journals, books, government studies, conference papers, conference presentations, and rigorous extension publications were classified as peer-reviewed papers. Non-peer-reviewed articles were classified as short extension releases, working papers, and industry articles. In total, there were 87 peer-reviewed and 23 non-peer-reviewed articles included in the analysis.

We make a distinction between peer-reviewed and non-peer-reviewed articles because of the differences in structure, content, and purpose of the articles. Peer-reviewed articles typically conduct rigorous economic or cost–benefit analyses that seek to answer a central research question. Non-peer-reviewed papers more often include broad overviews of current events in distillers’ grain markets, a basic explanation of a distiller’s related topic, or a relevant yet preliminary economic analysis. Peer-reviewed articles often analyzed the questions initially posed by non-peer-reviewed papers with greater detail which, in the case of distillers’ grains, often led to varying results and conclusions between studies. As a result, we found it useful to initially identify which papers were peer-reviewed and non-peer-reviewed but little reference is made to peer review status in the body of this analysis (see panel (b) Table 1). 

#### 3.2.3. Sectors Impacted by Distiller Grains

Articles often focused on the impacts that a particular distiller grain had on a specific industry. Many articles examined the impact of distillers’ grains or value-added products on more than one sector. We created and categorized all articles into three sectors—feeds (e.g., corn, soybeans, soybean meal, and other feed substitutes), livestock (e.g., beef cattle, dairy cattle, hogs, poultry, and other livestock), and other (e.g., ethanol, industrial gasses, and oil). Of the 110 articles included in the review, articles studied how distillers’ grains and valued-added products impacted only the grains sector (12), livestock sector (33), only other sectors (17), grains and livestock sector (33), grains and other sectors (12), livestock and other sectors (1), and all three sectors (2). Panel (c) in Table 1 shows which type of distillers’ grains or value-added products impacted one of the three primary sectors. 

Organizing the papers into these larger groups does limit some of the insights that are more obvious on a more granular level. For example, only six (8.5%) “livestock” papers include an analysis related to dairy. This is a relatively small amount given that distillers’ grains are a common component of dairy cattle rations. In general, studies show that DDGS can account for roughly 30% of dry matter in a lactating cow diet without reducing milk yield [14] but is generally between 10 and 15%. Higher levels have been known to impact the level and quality of milk fat [15]. This may be because dairy cattle primarily use distillers’ grains as a protein source and more easily substitute distillers’ grains with other high-protein products when costs increase. The heavy skew toward beef cattle and hogs in this research can also be attributed to the regions of the United States where distillers’ grains were studied in the publications, primarily from the Plains and Midwest. States in these regions skew heavily toward beef cattle and hog production and, therefore, tend to focus on those industries in their analyses.

#### 3.2.4. Location of Study

Papers examined distillers’ grains and value-added products in 15 different countries/regions worldwide (see panel (d) in Table 1). However, most of these papers focused on impacts within the U.S. distillers’ grains and value-added markets. Only DDGS was studied outside the U.S.

#### 3.2.5. Market Supply Chain

Papers were classified by whether their impact or analysis focused on the domestic market, export market, or both (see panel (e) of Table 1). In total, 80 of the 110 articles focused exclusively on domestic markets, and 30 incorporated international and export markets in some manner. Many of the domestic papers were published by universities based in the Great Plains and Midwestern regions. The 30 articles that incorporated international and export markets primarily focused on DDGS because DDGS are transportable, storable goods in comparison to MDGS and WDGS, which can only travel a limited radius. None of the export market-focused papers explored value-added goods. This is likely because value-added distillers’ grains were recently introduced, and many of the papers reviewed predate their arrival to the market. 

#### 3.2.6. Types of Distillers’ Grains

Articles primarily focused on analyzing one type of distillers’ grains or value-added coproduct—DDGS (87), MDGS (0), WDGS (3), and Value-added (1). Very few papers focused on more than one coproduct—two products (11) and three products (8). Most of the papers focused on DDGS as it is a more transportable product that comprises a much larger share of the distillers’ grain market than MDGS, WDGS, and value-added products (see columns in Table 1). From 2016–2020, the average proportion of distillers’ grain production in the United States was 53%, 12%, and 35% for DDGS, MDGS, and WDGS, respectively [16]. The transportability of DDGS versus MDGS and WDGS makes DDGS more relevant to a national or global audience whereas WDGS tends to be more focused on regional use among livestock producers. Value-added coproducts are relatively new to the coproduct market and thus little information is publicly known about their production, use, and market share; thus, very limited research has been conducted.

## 4. Results

The distinctions discussed above—such as types of distillers’ grains, commodities impacted by distillers’ grains, and location of study—help to better frame the existing state of the economic research related to ethanol coproducts. Broader similarities and differences between the papers are discussed in the body of the synthesis. In conducting this synthesis, we aim to summarize the economic findings of the existing research while identifying potential areas for future exploration. 

### 4.1. Cost Analysis

Articles in the cost analysis section primarily covered three broad categories: (1) ethanol plant cost structures [17,18], (2) coproduct generation [6,19], and (3) environmental costs of ethanol/distillers’ grain production [20]. Publication dates ranged from 2006 to 2016, with all but one paper being published before 2010 [18]. All studies focused on distillers’ grain production in the United States.

The ethanol plant cost structure studies centered around how ethanol plants use coproducts to offset production costs [17] and how variations in coproduct production alter the costs faced by ethanol plants [18]. Results suggest that ethanol plants market coproducts to offset ethanol production costs, but technological barriers to improving product quality and universality—resulting in distillers’ grains with high phosphorus content and corn germ and fiber that is indigestible to nonruminants—limited the efficacy of coproducts in reducing costs to plants [17]. Further, ethanol plants were found to change their coproduct mix in response to price signals [18]. For example, if export demand is weak and local market demand is strong, ethanol plants shift a greater percentage of their distillers’ grain production to wet distillers’ grains to reduce costs and increase coproduct profit margins. 

The coproduct generation studies focused on future coproduct generation rates [6] and pelletized distillers’ grain production [19]. They found that since the coproduct production process was heavily dependent on external market forces for future production levels, ethanol plants needed to pursue research focused on value-added products to diversify bioethanol revenue streams and hedge against potential adverse market conditions [19]. Pelletized distillers’ grains were one value-added product examined, the production of which was deemed a cost-effective process only in plants benefiting from the economies of scale [6]. Pelletized products create a product that is more consistent and customized to an operation. For example, in pelletized distillers’ grains, the fiber length and fat content can be controlled which makes them more useful in the diet formulation of dairy cattle. 

Finally, distillers’ grains were found to reduce the economic costs of ethanol production by diminishing the land use impact of ethanol’s use of corn [20]. Distillers’ grains can replace a certain percentage of the corn in a livestock ration. This can lead to a reduction in the total acres planted for corn thus reducing the total land and environmental impacts attributed to livestock production. The level of this reduction depends on the nutrient profile of the distillers’ grains, how much distillers’ grains are included in the livestock ration, and additional coproducts created. Overall, plants selling DDGS were more effective at offsetting their environmental impact than ethanol plants that did not market any coproducts and instead privately disposed of the product. 

### 4.2. Cost–Benefit Analysis

Cost–benefit analysis studies constituted the largest portion of the distillers’ grains economic literature in this review and featured a greater variety of locations and livestock types than other sections. This variety is likely attributable to the fact that the cost–benefit analysis papers mostly included economic/cost–benefit analyses as complementary components to more central research questions, typically the efficacy of feeding distillers’ grains to various livestock types. In other words, these economic analyses were largely budgeting exercises with different feeds within rations. These were included since some measure of the costs and benefits of distillers’ grains was included in each study. 

Thirty-two papers featuring nine different countries—Brazil [21], Bulgaria [22], Cuba [23], Egypt [24,25,26,27,28], Hungary [29], India [30,31], Philippines [32], Portugal [33], and the United States [34,35,36,37,38,39,40,41,42,43,44,45,46,47,48,49,50,51,52,53]—comprised the cost–benefit section of the review. 

The studies explored the value of feeding distillers’ grains ranging from DDGS to WDGS to high-fat and low-fat DDGS in beef cattle [26,35,36,40,41,49,53], dairy cattle [22,31,37,39,42,50], dairy buffaloes [32], fish [25,27,29,33,34,51], goats [30,52], poultry [23,28,38,44,46,47,48] hogs [21,43,45], and rabbits [24]. The papers examined distillers’ grains’ feeding value through the lens of animal performance analyses and models of representative operations. They then compared the cost of including distillers’ grains in animal diets versus the value of the changes in animal performance when fed distillers’ grains. Overall, the studies found distillers’ grains to be cost-effective, performance-enhancing, and profit-improving feed supplements and/or substitutes in beef cattle and mostly neutral responses in other species. However, a few studies found distillers’ grains to hurt livestock operation profitability relative to alternative feeds [26,43].

A significant shortcoming with these articles is that most use budgeting to justify economic usefulness. These studies can provide users with the development of larger market models that examine how the efficacy of feeding new and different distillers’ coproducts to unique livestock types can change market demand and supply. In this way, they can provide a valuable contribution to the existing literature by revealing what combinations of distillers’ grains type, location, and livestock type are most viable in the marketplace and, therefore, hold the most potential for more complex economic analyses. 

### 4.3. Demand Analysis

The demand analysis research in this literature review was divided into four broad categories: (1) contextual demand [54,55,56], (2) livestock demand [57,58,59,60,61,62,63,64], (3) location demand [65,66], and (4) potential demand [67,68,69,70]. The categories were determined by the primary research question from which ethanol coproduct demand was analyzed.

The contextual demand papers focused on distillers’ grain demand in response to external market forces, such as the COVID-19 pandemic, increased export demand, and ethanol production incentives [54,55,56]. All three articles were published after 2019 and emphasized the extent to which distillers’ grain markets rely on exogenous variables. More specifically, distillers’ grain prices and demand were found to be supported by increases in ethanol production, export opportunities, and storability, all of which helped the coproduct markets to rapidly recover from the initial impacts of COVID-19 [55,56].

Articles centering around the demand for distillers’ grains from livestock focused primarily on cattle, hogs, and general livestock markets [57,58,59,60,61,62,63,64]. The eight articles broadly concluded that feeding distillers’ grains is a viable method for increasing returns to livestock operations and pointed to nutritional variability, cost of transportation, corn prices, and feeding methods as factors that have significant impacts on the demand for and efficacy of feeding distillers’ grains. Demand for distillers’ grains from livestock operations was found to be sluggish to adjust to changes in these influencing factors [63]. In other words, only about a fifth of the long-run response to a change in the prices of feed grains was found to occur in the same marketing year as the price change [63]. Six of the articles were published before 2012, while two were published after 2014. Thus, the articles published before 2012 were written when the variability and nutritional characteristics, and storability of distillers’ grains were more prominent. Additionally, six of the eight studies only explored the demand for DDGS, while two expanded the analyses to include MDGS and WDGS. Given the increased visibility of distillers’ grains in the marketplace and the increased heterogeneity in product type and characteristics, there is little understanding of how the factors affecting the demand for distillers’ grains from livestock operations have evolved.

The two location demand papers examined factors influencing distillers’ grain production in Indiana and the United States as a whole [65,66]. Results included the prediction that livestock producers in Indiana and the United States would, overall, have ready access to distillers’ grains in the coming years [65]. The importance of including coproducts in ethanol plants’ life cycle analyses was also stressed [66]. Both location-based demand papers were published before 2012 and focused only on DDGS, indicating the need for updated and expanded additions to the economic research in this area to further explain the impact of location on coproduct demand. Without location-specific studies, the demand structures of ethanol plants outside the major production zone of the Midwest remain unclear. An unclear understanding of location’s impact on distillers’ grain demand, therefore, leaves researchers unable to analyze the impact of location-specific market shocks. 

The future demand for ethanol coproducts primarily focused on DDGS characteristics and market forces shaping its potential demand [65,67,68,69,70]. Feed quality heterogeneity and levels of per-animal consumption were topics of concern in the earlier articles while the prospect of demand outpacing supply was the focus of more recent articles [67,68,69,70]. Four of the five studies focused exclusively on the future of DDGS demand [65,67,69,70], while one study explored the viability of the market potential of extracting corn oil from DDGS for biodiesel production [68]. The outlook for ethanol coproducts demand was deemed positive. 

### 4.4. Economic Impact Analysis

Studies exploring the economic impacts of ethanol coproducts primarily did so through three lenses: (1) impacts on grain markets [71,72,73,74,75], (2) impacts on livestock markets [76,77,78,79,80] and (3) environmental impacts [81,82]. 

In the studies examining the impact of distillers’ grains on grain markets, the primary focus was the effect of distillers’ grain production on grain market structures and prices—specifically corn [74] and wider feed grain markets [71,72,73,75]. Papers analyzing the relationship between distillers’ grains and corn markets found that the onset of widespread ethanol production changed the directional flows of corn in the United States. In other words, corn flowed in-state to ethanol plants and then primarily out-of-state in the form of ethanol and distillers’ grains [74]. This changed the domestic supply dynamics of feed by altering what products were available and where. Studies exploring the broader relationships between distillers’ grain prices and feed grain prices found that correlations between general feed ingredient prices and crude oil prices increased dramatically since wide-scale ethanol production began [73]. This was because feed and fuel markets became more intertwined as corn was directed toward both animal feed and fuel ethanol markets. Because of corn’s unique status as both a livestock and fuel feedstock, the fact that the coproduct of corn ethanol production, distillers’ grains, could be fed to livestock in place of corn helped temper the inflationary effect of increased ethanol production on grain prices [71,72,75]. DDGS were the primary coproducts of focus in the grain impact analysis research. Given the changes in ethanol and coproduct production over the past decade, and the increased quantity of grains allocated toward that production, updated analyses of the impacts of ethanol coproducts on grain markets would fill a currently unaddressed gap in the literature. 

Studies centering around the impacts of ethanol coproducts on livestock markets all focused on DDGS in their analyses. Additionally, the studies found that while increased ethanol production increased general feed costs for livestock producers DDGS, for operations that were able to secure them, helped to reduce the negative impacts of higher feed prices and provided economic and nutritional benefits not present in previously used feedstuffs [76,77,78,79,80]. Biofuel mandates were found to encourage additional crop production and discourage livestock production in most global regions, especially for non-ruminant livestock that benefit less from ethanol coproducts [79]. All three studies were published in 2012 or before, meaning the analyses were unable to account for the many developments in the types and quality of ethanol coproducts over the past decade. These changes in ethanol coproducts have undoubtedly impacted the livestock industry, but the nature of those impacts is unknown. 

The studies exploring the economic and environmental impacts of ethanol coproducts found that excluding coproduct production from economic analyses of ethanol plants alters the results of biofuel mandates in systematic ways [81]. Models including coproducts show smaller changes in the production of cereal grains and larger changes to the production of oilseeds in the US and EU than models excluding coproducts [81]. Additionally, feeding coproducts to livestock was found to reduce the environmental footprint of ethanol plants relative to gasoline [82]. As with many of the previously discussed studies, the environmental and economic impact study only accounts for DDGS and WDGS in their analyses. The economic and environmental impacts of recent more specialized ethanol coproducts, such as pelletized distiller grains, have not been examined but could be assessed relative to the cost of corn.

### 4.5. Price Analysis

Price analysis research in this literature review fell primarily into three broad categories: (1) price discovery [83,84,85,86,87,88], (2) coproduct price effects [89,90], and (3) coproduct price relationships [91,92]. The price analysis section of the literature contained one of the largest proportions of recent publications of any section in this analysis, with most of the papers published since 2015.

The papers on price discovery in ethanol coproduct markets all focused exclusively on DDGS in the United States. In general, the articles found nutritional composition [83], location [84], and corn and soybean meal prices to be the most important determinants of DDGS prices [87]. The valuation of nutritional components varied by the type of livestock demanding DDGS. For example, the cost savings did not increase for hog operations as the crude level protein of DDGS increased and as DDGS was included at higher levels [85]. Given the recent development of high protein distillers’ grains as a separate product from DDGS these conclusions may not continue to hold. Further, given the relative recency of most of these studies, future contributions to the literature would have the greatest impact by diversifying the product types and locations examined. Price discovery analyses beyond DDGS and the United States would help to better frame the pricing structures of ethanol coproducts around the world.

The two price effect papers examined ethanol coproduct prices through disparate lenses: one explored the effects of a decline in DDGS prices, while the other analyzed the effects of changes in corn prices on DDGS. The first highlighted the risk of declining DDGS prices without declines in corn or soybean meal prices, which, ceteris paribus, would shrink ethanol plants’ profit margins [89]. Increases in corn prices, on the other hand, were found to lead to increases in DDGS prices—the production of which did not constitute a large enough share of the livestock feed market to offset corn price increases [90]. Both studies explored only DDGS prices in the United States, so suggestions for potential contributions to the literature mirror those provided above.

In the two price relationship papers, the authors examine spatial and time-varying price relationships between DDGS, corn, and soybean meal prices. Corn and soybean meal were found to contribute to each other’s prices, and corn was found to act as the largest contributor to uncertainty in DDGS prices [91]. DDGS prices were not found to influence corn and soybean meal prices [92]. Both studies examined the U.S. market and only examined DDGS prices. It is assumed that broad conclusions are likely to be similar between any individual ethanol coproduct, corn, and soybean prices. However, the effects of specialization and differentiation of ethanol coproducts on price relationships and whether product differentiation strengthens or weakens the relationship between corn and soybean meal prices are unknown. 

### 4.6. Risk Management

Seven studies comprised the risk analysis section of the literature review and explored topics primarily relating to cross-hedging, futures contracts, and transaction costs [93,94,95,96,97,98,99]. The studies were published from 2008 to 2019. 

The overall scope of risk management studies in the ethanol coproduct domain is limited. Only North American studies were found and analyzed in this literature review, and each study focused on DDGS for risk management. The studies came to differing conclusions regarding the potential effectiveness of cross-hedging DDGS with corn and soybean meal prices and, similarly, the potential efficacy of a DDGS futures contract in mitigating price risk. Given the fact that the attempt to create and maintain a DDGS futures contract failed, the studies following the failed contract more readily admit the difficulty of cross-hedging DDGS with corn and soybean meal, while those published before the introduction of the contract are more optimistic about its potential.

### 4.7. Techno-Economic Analysis

The techno-economic analysis papers in this literature review are categorized into two groups: (1) papers exploring the economics of converting distillers’ grains to biofuel [100,101,102,103] and (2) using novel processes to enhance distillers’ grains’ production or value [3,104,105,106,107,108,109,110,111]. Their publication dates ranged from 2006 to 2021, and all studies were based in the United States. 

Studies exploring the economic feasibility of using distillers’ grains for additional fuel production generally explored the costs and benefits of modular engineering processes for converting distillers’ grains into biofuels or biogasses. For the most part, converting distillers’ grains to fuel was found to be energy efficient and technically feasible, but the economic practicality of the processes was highly dependent on economies of scale and prevailing market prices. All papers ignored the cost of disposal of the non-edible residual distiller grains after the biofuel process as all feed is not converted to biofuel. As a result, no definitive economic conclusion was found across all four papers. Papers exploring enhancements in distillers’ grain production and/or value primarily analyzed how to produce distillers’ grains more efficiently or how to extract additional value out of distillers’ grains [105,106,107,110]. These research studies also came to differing conclusions about the efficacy of these processes. Notably, none of these papers examined the negative impact this could have on the livestock industry if products were changed or feed availability was altered. 

In general, the studies exploring enhancements to the distillers’ grain production process itself found those enhancements to be feasible [104,105,106,107,108], while the research analyzing processes producing new products was less definitive [3,105,109,111]. All papers conducted their analysis in the United States market. There is little research on whether international market players on either the ethanol or livestock production side would value these processes differently. 

### 4.8. Trade

Papers focused on international trade and production of ethanol coproducts were in two primary categories: (1) United States coproduct exports [112,113,114,115,116,117,118,119] and (2) international coproduct production [120,121,122,123]. The publication years ranged from 2008 to 2019 and all focused on the export of DDGS. Given the low transportability of MDGS and WDGS, the focus on dried distillers’ products was less exclusionary than some of the previous studies in other sections. Differentiation exists within dried distillers’ productions in the form of de-oiled, high protein, and pelletized products, though none of these products were explored in the studies. 

Papers that focused on U.S. exports of ethanol coproducts centered around potential export demand and export progress. Export demand from China was a primary focus [112,113,114,115,116,117]. The primary determinants of export demand were found to be the importing country’s meat production, technical barriers to trade, tariffs, and US ethanol production [119]. Overall, export outlooks were deemed positive, with quality heterogeneity concerns identified as one of the most significant barriers to increased export activity [112,113,114,115,116,117,118]. Finally, China was found to be a major player in the market, with the ability to almost drive DDGS to demand singlehandedly when fully participating in the marketplace [113,114,115,116,117,118]. This was highlighted by the fact that China has tended to break contracts and commitments on current purchase agreements but will then enter the market several months later at a lower price. No studies have examined the impact of these trade dynamics and violations. 

Trade and international use of coproduct papers with a specific focus on coproduct production in international markets explored the implications of ethanol and coproduct production in Argentina [119], the European Union [120], and South Africa [121,122]. Studies have found that ethanol production increased the competitiveness of feed industries in the countries and regions explored. The benefit of feed competitiveness was a result of DDGS reducing phosphorus contents of feed rations and reducing ration costs—both not available without DDGS [119]. No studies examined other ethanol coproducts nor how internationally produced DDGS differ from those produced domestically.

## 5. Discussion

The previous sections have reviewed and synthesized the existing literature on economic studies in the ethanol coproducts space. This has shown several gaps where economic studies are needed. We review what additional lines of research could be investigated to better align research with the current direction of the ethanol coproduct industry.

### 5.1. Value-Added Distillers’ Grains

Value-added distillers’ grain products include high protein, pelletized, and de-oiled distillers’ grains, among many other products. In some cases, these value-added coproducts have fundamentally shifted the distillers’ grain landscape by providing features distinct from traditional ethanol coproducts. Most distillers’ grains were sold without extracting the corn oil before 2012. Then, ethanol plants began to extract oil and sell do-oiled distiller grains and corn oil separately. Now, de-oiled distillers’ grains are nearly ubiquitous in the marketplace and are generally sold under the traditional DDGS, MDGS, and WDGS umbrellas [124]. Livestock producers have frequently suggested that the removal of corn oil fundamentally changed the nutritional value of the product but the market price reflected an oiled product. Just recently, a large study has been conducted which showed that, at least for beef cattle in the Northern Plains, performance between full fat and de-oiled products was similar [125]. Little is known about how corn oil impacts ethanol plant profitability and how the pricing of distillers’ grains should be changed if at all, given changes in the underlying distillers’ grain product.

Pelletizing dried distillers’ grains is not known to change the nutritional composition of the product [34] but offers logistical benefits to traditional distillers’ grains in their ease of transportation, storage, and feeding [4]. It also allows distiller grains to be modified in fat and fiber length which makes them able to be used in other feed rations, notably with dairy cattle. Understanding what price premium these logistical benefits earn in the marketplace, how that premium compares to the cost of pelletizing distillers’ grains, and how much would this pull away from the traditional products are unknown.

High protein distillers’ grains have the greatest potential to impact the traditional distillers’ grain market however they change the nutritional composition. These grains are produced using pre-treated hydrolyzed distillers’ grains to increase protein content, or they can be made by separating the fiber from traditional distillers’ grains to produce higher-fat, higher-protein products—among many other production methods [3,104,108]. Some research is being conducted to understand how the subsequent nutritional change affects different livestock types and if those variations in feeding value are consistent across livestock types. However, little is known if the cost of producing high protein distillers’ grains versus the premium or discount the products received in the marketplace is profitable for ethanol plants, and how livestock producers perceive and are willing to pay for a lower protein feed. Very few economic studies have examined these sub-markets that have fundamentally changed what distillers’ grains are produced by ethanol plants. As not all plants have these different value-added operations, aggregating prices and quantities across space and time can create significant estimation challenges. All future studies examining value-added processes should consider the value of the additional product made and the value of the remaining components in the new commodity distillers’ grains.

### 5.2. Governmental Regulations and Industry Challenges

Few topics of potential interest to distillers’ grain economic research seem to undergo as much change, or face the potential of change, as the ever-evolving government regulations facing the ethanol and distillers’ grain industries. Despite the continual specter of new or adjusted regulations and, concurrently, altered industry structures, few studies have examined the economic impact of these regulations directly on the distillers’ grains industry. Most of the studies that do explore governmental regulations infer these impacts through the lens of regulations on the ethanol industry [126]. For example, studies analyzing the impact of minimum fuel blending requirements inherently incorporate an impact on distillers’ grains regardless of whether they are explicitly included in the analysis. 

Regulations that increase ethanol output will increase distillers’ grain output, while those that decrease ethanol output will result in a commensurate decline in distillers’ grain availability. These studies, therefore, occupy a well-explored section of the literature, even if more attention could be paid to the exact economic impacts of these increases or decreases in distillers’ grain availability. However, areas with little-to-no existing economic research include regulations that directly impact distillers’ grain products, such as nutrition or quality standards. 

Currently, there are no nutritional standards to differentiate distillers’ grains besides moisture content. Some ethanol plants will self-report nutritional composition on their website on either a one-time or ongoing basis. The variation in nutritional content between plants can vary significantly making it difficult for livestock producers to determine the value of the product they are purchasing as well as changes in animal performance from nutritional variation. If livestock producers knew the minimum protein, fat, and oil contents of various distillers’ grain products, for example, they could more effectively measure the costs of purchasing the products versus the value they would provide to their operation. On the other hand, mandating minimum nutrition or composition standards may impose new costs on ethanol plants. A full cost–benefit analysis would likely need to consider how any new regulations would outweigh the benefit to the marketplace. Quality standards pose a similar issue for the distillers’ grains industry. These standards would likely include rules relating to maximum levels of mycotoxins or phosphorus and require ethanol plants’ products to meet those standards. While these standards would not likely impact the feeding value of distillers’ grains in terms of nutritional composition, they would guarantee a safe product to end-users, thus limiting animal health issues and a consistent feeding product. As the industry continues to evolve and products are further refined to extract additional value, likely some effort is needed to define what adequately constitutes various distillers’ grain studies examining the potential impact of these regulations would provide much-needed insight into the market. 

### 5.3. Changes in Location and Commodities

Expanding the scope of international research is another need. Given the scale of both the ethanol and livestock feeding industries in the United States, many studies have already explored the basic economics of distillers’ grains from both the ethanol plant and livestock producer perspectives. Whether these economics are the same internationally—given unique market structures, differing end users, and varying feed inputs—is largely unexplored beyond basic cost–benefit analyses in lower-visibility industries such as aquaculture and alternative poultry operations. Further international research on distillers’ grains may help to uncover areas in which domestic ethanol plants could expand their reach. Conversely, adverse outcomes to international distillers’ grain markets may portend an unfriendly future domestically. Either way, research in this area would help to better shape the state of the global distillers’ grains industry. Any of the research categories found in this literature review would be useful angles from which to explore international distillers’ grain topics. 

Future research studies would also do well to branch out beyond analyses that primarily explore cattle and hogs. Given cattle and hogs’ importance to the distillers’ grains industry, the heavy focus on those species in ethanol coproduct research is understandable, but it has resulted in several gaps in the literature. Is there a viable path forward for ethanol coproducts in the dairy, poultry, and aquaculture industries? Are there species currently underutilizing distillers’ grains from a profit maximization perspective, or will the market fail to expand beyond cattle and hogs? These studies would likely consist primarily of cost and demand analyses and would help to determine the long-term demand for distillers’ grains in each specific segment.

## 6. Conclusions

In total, the 110 papers analyzed in this review and synthesis covered a broad swath of economic topics in the distillers’ grains domain. Much of the research covered in this literature review appeared during the ethanol boom of the first decade of the 2000s and shortly thereafter. Overall, research has found distillers’ grains to be cost-effective, profitable, and potentially impactful products with the potential for significant market growth. Still, many research questions remain unanswered. 

Based on this review and synthesis, we provided areas where additional economic research could be conducted. This consisted of (a) value-added distiller grain products, (b) government and industry regulation, and (c) a change in focus of location and commodities. Understanding the market and the factors driving change, such as protein content or oil type, are fundamental to conducting basic economic analysis which often requires aggregating prices and quantities across space and time. Not accounting for these factors can create significant estimation challenges. It also calls into question whether current prices and quantities being reported by USDA are sufficient to conduct such analyses. 

Overall, the state of the industry has moved well beyond the existing academic literature. Most of the studies examined were published more than five years ago and very few differentiated between the changes in distiller grains such as high protein and corn oil extraction. Given these significant advancements in the domestic and international distillers’ grain industries, more up-to-date research is needed to verify prior results and examine new developments in the distillers’ grains domain. Such research would better inform policy, planning, and decision-making from both ethanol plant and livestock producer perspectives and would more accurately reflect today’s distillers’ grains industry.

## Figures and Tables

**Table 1:** Topics examined, peer review status, sector of importance, market supply chain, and geographic location by type of ethanol coproduct analyzed.

	One Product				Two Products	Three Products	Total
	DDGS	MDGS	WDGS	Value-Added			
Panel (a): Primary Topic							
Cost Analysis	3	0	0	1	1	0	5
Cost–Benefit Analysis	30	0	1	0	2	0	33
Demand Analysis	12	0	0	0	3	2	17
Economic Impact Analysis	9	0	0	0	1	2	12
Price Analysis	9	0	0	0	0	1	10
Risk Management	5	0	0	0	2	0	7
Techno-Economic Analysis	9	0	2	0	2	0	13
Trade	10	0	0	0	0	3	13
Panel (b): Peer Reviewed Status							
Non-Peer Reviewed	17	0	0	0	2	4	23
Peer Reviewed	70	0	3	1	9	4	87
Panel (c): Sectors Examined							
Feed	11	0	0	0	0	1	12
Livestock	24	0	1	0	3	5	33
Other	11	0	2	1	3	0	17
Feed + Livestock	27	0	0	0	4	2	33
Feed + Other	11	0	0	0	1	0	12
Livestock + Other	1	0	0	0	0	0	1
Feed + Livestock + Other	2	0	0	0	0	0	2
Panel (d): Geographic Location							
Argentina	1	0	0	0	0	0	1
Brazil	1	0	0	0	0	0	1
Bulgaria	1	0	0	0	0	0	1
Canada	0	0	0	0	1	0	1
China	1	0	0	0	0	0	1
Cuba	1	0	0	0	0	0	1
Egypt	6	0	0	0	0	0	6
European Union	1	0	0	0	0	0	1
Hungary	1	0	0	0	0	0	1
India	1	0	1	0	0	0	2
Philippines	1	0	0	0	0	0	1
Portugal	1	0	0	0	0	0	1
South Africa	2	0	0	0	0	0	2
United States	68	0	2	1	10	7	88
Worldwide	1	0	0	0	0	1	2
Panel (e): Market Supply Chain							
Domestic	62	0	2	1	10	5	80
Export	23	0	1	0	1	2	27
Domestic & Export	2	0	0	0	0	1	3

Source: Authors’ calculations. Note: There are a total of 110 studies; column sums of articles are the same between panels at only DDGS (87), only MDGS (0), only WDGS (3), only value-added (1), two products (11), and three products (8); two products include DDGS + MDGS (4) and DDGS + WDGS (7); three products include DDGS + MDGS + WDGS (8); value-added products include corn oil (2) low/high fat DDGS (2), hydrolyzed DDGS (1), and pelletized DDGS (1).

## Data Availability

Data is available upon request from the corresponding author.

## Supplementary Materials

The following supporting information can be downloaded at: https://www.mdpi.com/article/10.3390/ani14111551/s1, Table S1: The master table of articles and classification.
